# Determination of the anti-yeast activity of Lactobacillus spp. isolated from traditional Iranian cheeses *in vitro* and in yogurt drink (Doogh)

**DOI:** 10.1038/s41598-020-63142-0

**Published:** 2020-04-14

**Authors:** Saeid Afzali, Mohammad Reza Edalatian Dovom, Mohammad Bagher Habibi Najafi, Mostafa Mazaheri Tehrani

**Affiliations:** Food Science and Technology Department, Agriculture Faculty, Ferdowsi University of Mashhad (FUM), Mashhad, Iran

**Keywords:** Biotechnology, Microbiology, Bacteria

## Abstract

The objective of this study was to determine the anti-yeast effect of Lactobacillus spp. isolated from two popular Iranian cheeses known as Lighvan and Motal against food spoilage yeasts known as *Rhodotorula mucilaginosa*, *Saccharomyces cerevisiae* and *Kluyveromyces lactis*. Twenty strains of Lactobacillus were selected from Motal (16 isolates) and Lighvan cheeses (4 isolates). Anti-yeast activity was studied by Agar Spot and Well Diffusion Assay. Effect of pasteurization on inhibitory compounds was also investigated. Results showed that two strains of *Lactobacillus brevis* (M4 and M2) exhibited the highest anti- yeast activity in aforementioned methods, as well as tolerated pasteurization. M4 and M2 strains were inoculated into Doogh (a fermented yogurt drink) at two levels (10^6^ and 10^8^ cfu/ml). All samples were incubated in three storage temperatures (4, 25 and 37 °C) and were then examined for microbial parameters (Mold and yeast counts, Coliform, *E. coli* and *Staphylococcus aureus*) at specific intervals. Sample with M4 (10^6^cfu/ml) showed superiority rather than control sample in microbial point of view. At temperatures of 25 and 37 °C, inoculated samples were not contaminated up to day- 21 and day-14, respectively. The propionic acid content for M4 and M2, was 14576.11 and 11697.3 ppm, respectively. Results indicate that incorporation of strain M4 (*Lb. brevis*) at a level of 10^6^ cfu/ml can potentially postpone the yeast spoilage in Doogh and prolong the stability of this product. In conclusion, these strains had the highest activity against experimented yeasts.

## Introduction

Fermented commodities present a crucial effect in human dietary all over the world owing to their beneficial effects on health. Fermentation is one of the ancient and most commercial approach applied for food preservation. The primary precedent exhibit that mankind was consuming ‘acidified or fermented milk’ dated to about 2000 years ago^1^.

Yogurt drinks are known in the Middle East with various titles such as Lassi, Doogh, Yogurt and Ayran, and in Scandinavian countries, as acidophilus milk, Viili (a ropy, fermented milk that originated in Scandinavia, which is claimed to have various functional benefits and health-improving potential which is popular in Finland),^2^. Doogh is a fermented dairy product that is obtained from the mixing of yogurt with water and some other additives such as salt and mint^2^. Presently, Doogh has gained acceptable popularity and wide consumption in Iran and per capita consumption as well as industrial production has achieved noticeable growth in recent years. Acceptability of Doogh is not only related to the desirable sensory attributes but also to healthy fermented drink which make it accepted as national drink in Iran. Doogh has also gained high popularity in other countries according to the literature^3^.

Fungal spoilage of food and feed is a universal issue. It has been estimated that almost 5–10% of world food production is lost as a result of fungal spoilage with economic losses^4^. Furthermore, the growth of particular fungi on food can lead to the production of mycotoxins, which are known to be toxic to humans and animals^5^.

Food spoilage attributable to yeasts represents a significant problem for dairy and beverage industries. Because of ability of yeasts to survive at low temperatures and acidic pH, yeasts cause frequent food spoilage in yogurt, cheese and fruit juices^6^.

Three types of yeast, which cause foamy, bottle swelling and undesirable flavors in yogurt and yogurt drink are, *Saccharomyces cerevisiae, Kluyveromyces lactis* and *Geotrichum* sp.^7,8^. The risk of contamination is very high for homemade Doogh, particularly by *Kluyveromyces* spp. and *Saccharomyces* spp.^2^. The presence of *Kluyveromyces lactis*, *S. cerevisiae* and *Geotrichum* spp. in some drink yogurt has been reported at low levels^9^. Yeasts are one of the largest groups of microorganisms that spoil non-alcoholic beverages, juices, dairy products and fermented beverages such as Doogh with side-effects such as CO_2_ and acid production^10^.

Lactic acid bacteria (LAB), as well as their metabolites, have traditionally been used as natural preservatives in food and feed. The protective effects caused mainly by the formation of LAB metabolites such as organic acid, hydrogen peroxide, competition for nutrients and the production of antimicrobial substances^11^. Lactic acid bacteria produce various antimicrobial compounds, such as lactic and acetic acids, as well as hydrogen peroxide, formic acid, propionic acid, and diacetyl^12^. Lactic acid bacteria are either naturally occurring in the food or intentionally added to food formulation. They are generally recognized as safe (GRAS) or even improve human and animal health. It is estimated that 25% of the European diet, as well as 60% of the diet of many developing countries, is made up of fermented foods^11^.

Lactic acid bacteria are also used as a starter in the manufacture of dairy products such as acidophilus milk, yogurt, buttermilk, and cheese^13^. Our previous study indicated that some isolated lactic acid bacteria showed antibacterial activity^14^.

The aim of this study was to investigate the anti-yeast effect of Lactobacillus spp., isolated from Iranian raw milk Lighvan and Motal cheeses on *Rhodotorula mucilaginosa, Saccharomyces cerevisiae* and *Kluyveromyces lactis* as indicators for food spoilage yeasts in doogh both *in vitro* and *in situ*.

## Materials and Methods

Twenty strains of Lactobacillus spp. isolated from traditional Iranian cheeses (16 isolates from Motal cheese and 4 isolates from Lighvan cheese) (Table 1) previously identified by molecular techniques followed by sequencing the 16 S rRNA gene^14,15^ were screened for anti-yeast activity. *Rhodotorula mucilaginosa* (PTCC 5257), *Saccharomyces cerevisiae* (PTCC 5269) and *Kluyveromyces lactis* (PTCC 5185) were used as yeast spoilage indicators. In order to determine the *in vitro* anti-yeast activity of Lactobacillus strains in the culture medium, Agar Spot Assay with growing cells^16^ and Well Diffusion Assay with the cell-free supernatant^17^ were used.

**Table 1:** Results of the anti-yeast effect of Lactobacillus strains against *Rhodotorula mucilaginosa*, *Saccharomyces cerevisiae* and *Kluyveromyces lactis*, by Agar Spot method (diameter of the Inhibition hole in millimeters).

Cheese type	Genus and species	Sample code*	*Rhodotorula mucilaginosa*	*Saccharomyces cerevisiae*	*Kluyveromyces lactis*
			Average diameter of the halo	Average diameter of the halo	Average diameter of the halo
Motal	*Lactobacillus brevis*	M2	7.5*^a^ ± 0.707	6^ab^	5^ab^ ± 1.41
Motal	*Lactobacillus brevis*	M3	6^ab^ ± 1.41	5.5^ab^ ± 0.707	4.5^ab^ ± 0.707
Motal	*Lactobacillus brevis*	M4	7^a^	6.5^a^ ± 0.707	4.5^ab^ ± 0.707
Motal	*Lactobacillus brevis*	M5	6^ab^ ± 1.41	6.5^a^ ± 0.707	4^ab^
Motal	*Lactobacillus brevis*	M6	6^ab^ ± 1.41	0	3.5^ab^ ± 0.707
Motal	*Lactobacillus brevis*	M7	0	0	3.5^ab^ ± 0.707
Motal	*Lactobacillus brevis*	M8	5.5^ab^ ± 0.707	6^ab^	5^ab^
Motal	*Lactobacillus brevis*	M9	5^ab^	0	0
Motal	*Lactobacillus brevis*	M11	0	3.5^ab^ ± 0.707	4^ab^ ± 1.41
Motal	*Lactobacillus brevis*	M12	4^ab^ ± 1.41	4.5^ab^ ± 0.707	4^ab^
Motal	*Lactobacillus brevis*	M13	6^ab^	0	4.5^ab^ ± 0.707
Motal	*Lactobacillus casei*	M15	5.5^ab^ ± 0.707	5^ab^ ± 2.82	4^ab^ ± 1.41
Motal	*Lactobacillus plantarum*	M16	3.5^ab^ ± 0.707	5^ab^	4^ab^
Motal	*Lactobacillus plantarum*	M17	5.5^ab^ ± 0.707	6^ab^	2.5^ab^ ± 0.707
Motal	*Lactobacillus plantarum*	M18	6^ab^ ± 1.41	5.5^ab^ ± 0.707	0
Motal	*Lactobacillus plantarum*	M19	6.5^a^ ± 0.707	5^ab^	0
Lighvan	*Lactobacillus plantarum*	M8(Lighvan milk)	4^ab^	4^ab^	4.5^ab^ ± 0.707
Lighvan	*Lactobacillus plantarum*	LF52	5.5^ab^ ± 0.707	5^ab^	4^ab^ ± 1.41
Lighvan	*Lactobacillus plantarum*	LF55	5.5^a^ ± 0.707	6.5^a^ ± 0.707	6.5^a^ ± 0.707
Lighvan	*Lactobacillus plantarum*	LF56	7^a^ ± 1.41	5.5^ab^ ± 0.707	5.5^ab^ ± 0.707

*M stands for Motal cheese and LF stands for Lighvan fresh Cheese.

*All data (results) were obtained from subtraction of diameter of clear zone and diameter of the spot (4 mm) and reported in mm.

Non-similar alphabets in each column represent mean values at the level of α = 95% or (p-value < 0.05).

### Preparation of cell-free supernatant (CFS)

Lactobacillus strains were cultured in de Man Rogosa and Sharpe broth (MRS, Merck, Darmstadt, Germany) for 24 h at 37 °C and were then centrifuged at 9500 × g for 15 minutes. The supernatant was filter-sterilized (0.45 μm)^17^. Stock cultures of strains were prepared with MRS broth plus Gly (30%) and stored at −80 °C for long term-storage of studied strains.

### Well diffusion assay

The agar well diffusion assay was performed as reported by Yang and Chang (2010) with slight modifications. Briefly, plates of PDA (Merck, Darmstadt, Germany) containing 10^6^ CFU per 20 ml of agar medium of each yeast indicator and then placed in the incubator at 25 °C for 5 to 7 days.

Wells of 6 mm in diameter were punctured. Inside the wells, 100 μl of CFS was poured. In order to allow the CFS diffusion into the agar, the plates were preincubated in a refrigerator (4 °C) for 1 h and then incubated in 25 °C. Anti-yeast activity was assessed after 5 to 7 days, by measuring the diameter of the growth inhibition zone in millimeters^17^. All reported data are the difference between the diameter of the clear zone and the diameter of the well (8 mm) in mm. Experiments were done in duplicates.

### Influence of heat treatment on the inhibitory ability of CFS

In order to investigate the influence of pasteurization on the anti-yeast activity, the agar well diffusion assay was performed using pasteurized CFS. The CFS were subjected to low- temperature long time (LTLT) at 65 °C for 30 min in a water bath (Memmert, Germany). Untreated CFS was used as control. 100 μl of each supernatant was poured into each well of culture medium containing 10^6^ colonies of indicator yeast per 20 ml of PDA and then placed in the incubator at 25 °C for 5 to 7 days. The results were reported by measuring the diameter of the inhibition zone in millimeters and as an average of two replicates^16^.

### Agar spot method

To evaluate the anti-yeast activity by the agar spot assay, the method reported by Rouse *et al*. (2008) was used with slight modification. Briefly, each Lactobacillus strain was cultivated in MRS broth incubated at 37 °C for 24 h. Then, 5 µl were spotted on MRS agar plates incubated at 37 °C for 24 h. After this incubation time, the plates were overlaid with a soft PDA (0.75% w/v agar), previously inoculated with 10^6^ CFU/ ml of each yeast spoilage indicator (*Rhodotorula mucilaginosa*, *Saccharomyces cerevisiae*, and *Kluyveromyces lactis*), and incubated at 25 °C. Anti-yeast activity was assessed after 5 to 7 days, by measuring the diameter of the growth inhibition zone in millimeters^16^. Experiments were done in duplicates.

### Measurement of propionic acid production

Since propionic acid is considered one of the antifungal metabolites of LAB, it was measured in this study. The supernatants obtained from two strains -M2 and M4- (with the highest clear inhibition zone in well diffusion Assay) were passed through sterilization filter (0.45 μm). The amount of propionic acid produced was measured using HPLC equipped with RP-C18 column (3 μm particle size, 150 4.6 mm I.D., kept at 25 °C) and a UV–Visible diode array detector (DAD). The mobile phase consisted of 0.01 mol/ L KH_2_ PO_4_ buffer solution (pH = 2.60 adjusted with o-phosphoric acid), using an isocratic elution procedure with a flow rate of 1.5 mL/ min. Detection was performed at 210 nm for propionic acid^18^.

### Yogurt drink (doogh) production

Production of yogurt drink was performed according to the Iranian Standard No.2453.Two best-selected strains (M2 and M4: *Lb. brevis*) with the highest anti-yeast activity, were added as adjunct culture along with commercial yogurt starter (Micro Milk Company, 2100–1–10 Dosi) (1%) into pasteurized milk (90 °C,10 min) at 37 °C. The selected Lactobacillus strains were inoculated at two levels each(10^6^ and 10^8^ CFU/ ml), and a sample with no added adjunct culture was considered as control, then fermentation continued at 37 °C until pH reaches 4.6^19^. Water was then added to the yogurt in a ratio of 1:1 and was mixed with 1% food grade salt. Yogurt drink was then pasteurized at 63 °C, 30 min. and packed. All samples were stored at three different temperatures (4, 25 and 37 °C) and were then examined for microbial parameters (Mold and yeast counts, Coliforms, *E. coli* and *Staphylococcus aureus*) at specific intervals^20^.

Doogh production on an industrial scale was also conducted to evaluate the gas production in one-liter bottled Doogh in the dairy pilot plant. Doogh production was carried out in accordance with the Iranian National Standard Number. 2453^20^.

### Microbial evaluation

Study of microbial and physicochemical properties of Doogh including Mold & yeast, Coliforms, *E. coli*, *Staphylococcus aureus* and pH was performed during 60 days of storage at 4 °C in 10 day intervals, at 25 °C for 21 days in 7 day intervals and at 37 °C for 14 days in 7 day intervals^19^.

Coliform enumeration was done with the method of counting in the Most Probable Number (MPN method) after incubation of the inoculated broth medium at 30 °C^21^. Molds and yeasts were evaluated using Surface Method on YGC by colony counting method at 25 °C, and coagulase positive staphylococci were determined using Surface Method on Baird*-*Parker agar for 48 h at 37 C. This method is applicable in cases where the staphylococci are damaged or their number is low in the sample^22^. Counts were determined according to National Iranian Standard Numbers– 5486–1, 5234 & 5486–2, 997 and 6806^20–24^.

### Sensory analysis

Sensory evaluation was performed according to the Iranian National Standard No. 4691^25^. Sensory parameters including taste, texture, color and total acceptance were evaluated by 10 well trained food science and technology students.

### Statistical analysis

Data were analyzed in a completely randomized design in a factorial arrangement. The results were analyzed using the Mini Tab (Version 16) software and Excel charts were used to plot the data. The variables included number of strains (2 strains: M2 and M4), inoculum levels (at three levels), temperature and storage time. At 4 °C for 60 days of storage in 10 day intervals, at 25 °C for 21 days in 7 day intervals and at 37 °C for 14 days in 7 day intervals.

## Results

Results of the anti-yeast effect of 20 strains of Lactobacillus spp. isolated from traditional Motal and Lighvan cheeses against *Rhodotorula mucilaginosa*, *Saccharomyces cerevisiae* and *Kluyveromyces lactis* using Agar Spot method are presented in Table 1.

The highest halo diameter against *Rhodotorula mucilaginosa* was related to strain M2 (*Lb. brevis* KX572376 isolated from Motal cheese) with a diameter of 7.5 mm and the lowest halo diameters were obtained for strains M7 and M11 (*Lb. brevis* isolated from Motal cheese) (equal to zero, Table 1). Regarding the *Saccharomyces cerevisiae*, the highest halo diameter was related to strain M4 (*Lb. brevis* KX572378) with a diameter of 6.5 mm and the lowest one for strains M6, M7, M9, and M13. The highest anti-yeast activity against the *Kluyveromyces lactis* was detected for strain LF55 (*Lactobacillus plantarum* isolated from Lighvan cheese) with a diameter of 6.5 mm and the lowest one was found for the M9 (*Lb.brevis*), M18 (*Lb. plantarum*), and M19 (*Lb.plantarum*) with a diameter of zero.

*Rhodotorula mucilaginosa, Saccharomyces cerevisiae*, and *Kluyveromyces lactis* were inhibited by 18 (90%), 16 (80%) and 17 (85%) examined isolates, respectively. From these results, it is implied that *Saccharomyces cerevisiae* was characterized as the most resistant yeast among these indicators.

In Table 2 effects of indicators (yeast type) on the diameter of inhibition zone were shown. The most inhibitory effect as shown, is against *Rhodotorula mucilaginosa* (5.35 ± 1.77) and the least inhibitory effect on *Kluyveromyces lactis* (3.97 ± 1.18).

**Table 2:** Influence of yeast (indicator) type on the diameter of the clear zone of inhibition.

Indicator (Yeast type)	The average diameter of the halo
*Rhodotorula mucilaginosa*	5.35^a^ ± 1.77
*Saccharomyces cerevisiae*	4.77^a^ ± 1.46
*Kluyveromyces lactis*	3.97^b^ ± 1.18

In Table 3 results of the anti-yeast effect of CFSs of 20 Lactobacillus strains from traditional Motal and Lighvan cheeses were reported against *Rhodotorula mucilaginosa, Saccharomyces cerevisiae* and *Kluyveromyces lactis* using Well Diffusion Assay method, as well as the effect of LTLT pasteurization. The results were expressed as the mean of two replicates of the inhibitory zone diameter.

**Table 3:** The results of the anti-yeast effect of Lactobacillus strains on *Rhodotorula mucilaginosa* (R), *Saccharomyces cerevisiae* (S) and *Kluyveromyces lactis* (K) by Well Diffusion Assay method (diameter of the Inhibition halo in millimeters).

Indicators (Food Spoilage yeasts)		*R. mucilaginosa*		*S. cerevisiae*		*Klu. lactis*	
Genus and species	Sample code	25 °C	LTLT	25°C	LTLT	25°C	LTLT
*Lactobacillus brevis*	M2	7^ab^	6^ab^ ± 1.41	7.5^ab^ ± 0.707	7^ab^	6^ab^	4.5^ab^ ± 0.707
*Lactobacillus brevis*	M3	0	0	6^ab^ ± 1.41	0	0	0
*Lactobacillus brevis*	M4	7.5^ab^ ± 0.707	7^ab^ ± 1.41	9.5^a^ ± 2.12	6.5^ab^ ± 0.707	7^ab^	5^ab^ ± 1.41
*Lactobacillus brevis*	M5	6^ab^	0	0	0	0	0
*Lactobacillus brevis*	M6	5.5^ab^ ± 3.53	4^ab^ ± 1.41	5.5^ab^ ± 0.707	5.5^ab^ ± 0.707	4^ab^	3.5^ab^ ± 0.707
*Lactobacillus brevis*	M7	0	0	0	0	0	0
*Lactobacillus brevis*	M8	0	0	7^ab^ ± 1.41	0	5^ab^	4.5^ab^ ± 0.707
*Lactobacillus brevis*	M9	0	0	6.5^ab^ ± 0.707	4.5^ab^ ± 2.12	5^ab^ ± 1.41	0
*Lactobacillus brevis*	M11	5^ab^	0	6^ab^ ± 1.41	5^ab^ ± 1.41	4^ab^ ± 1.41	0
*Lactobacillus brevis*	M12	0	0	6^ab^ ± 2.82	0	0	0
*Lactobacillus brevis*	M13	6.5^ab^ ± 2.12	0	6.5^ab^ ± 2.12	0	4.5^ab^ ± 0.707	0
*Lactobacillus casei*	M15	6.5^ab^ ± 2.12	6^ab^ ± 1.41	3^ab^	0	4.5^ab^ ± 0.707	0
*Lactobacillus plantarum*	M16	8^ab^	0	5^ab^ ± 1/41	0	0	0
*Lactobacillus plantarum*	M17	7^ab^	6.5^ab^ ± 0.707	4.5^ab^ ± 0/707	4^ab^ ± 1.41	4^ab^	0
*Lactobacillus plantarum*	M18	0	0	0	0	0	0
*Lactobacillus plantarum*	M19	8^ab^	6.5^ab^ ± 0.707	0	0	0	0
*Lactobacillus plantarum*	M8(Lighvan milk)	7.5^ab^ ± 0.707	0	5^ab^	4^ab^	0	0
*Lactobacillus plantarum*	LF52	7.5^ab^ ± 0.707	7^ab^	0	0	0	0
*Lactobacillus plantarum*	LF55	7^ab^	0	8^ab^	0	5^ab^	0
*Lactobacillus plantarum*	LF56	7^ab^	6.5^ab^ ± 0.707	6.5^ab^ ± 0.707	0	4.5^ab^	4^ab^ ± 0.707

M stands for Motal cheese and LF stands for Lighvan fresh Cheese.

All reported data are the difference between the diameter of the clear zone and the diameter of the well (8 mm) in mm.

Non-similar alphabets in each column represent mean values at the level of α = 95% or (p-value <0.05).

Highest halo diameter against *Rhodotorula mucilaginous*, before LTLT treatment, belonged to M16 and M19 strains (*Lb. plantarum*) with a diameter of 8 mm inhibition zone. After subjecting to LTLT treatment, halo diameter decreased. However, strain M4 showed a clear zone of inhibition equal to 7.5 mm before LTLT which reduced to 7 mm following the LTLT. Thus, strain M4 has maintained its anti-yeast properties after LTLT. In eight out of 20 isolates (40%), the anti-yeast property has been observed after LTLT. The highest halo diameter against *Saccharomyces cerevisiae* related to M4 strain with an inhibition halo diameter equal to 9.5 mm before LTLT and decreased to 6.5 mm after LTLT treatment. Totally, 7 out of 20 (35%) isolates, were exhibited anti-yeast properties, a clear zone of inhibition, after heat treatment test. Also, *Kluyveromyces lactis* was inhibited by M4 strain maximally with an inhibition diameter of 7 mm before LTLT and reduced to 5 mm after LTLT. A total of 5 out of 20 isolates (25%), were exhibited anti-yeast properties following the heat treatment.

**Figure 1:**
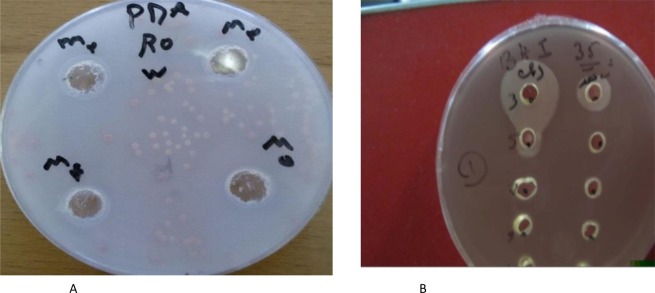
Well, diffusion assay against *Rhodotorula mucilaginosa* before heat treatment (LTLT) (**A**) and after heat treatment LTLT (**B**).

The diameter of the inhibitory zone before and after the LTLT treatment against *Rhodotorula mucilaginosa* is shown in Fig. 1.

In order to evaluate the effect of thermal treatment (LTLT) on supernatant of LAB, the supernatant was subjected to heat treatment. Results indicated that supernatant of strains retained their anti-yeast activity after heat treatment. With other words, clear zone of inhibition completely maintained with no difference in diameter following the heat treatment.

### Propionic acid content

**Figure 2:**
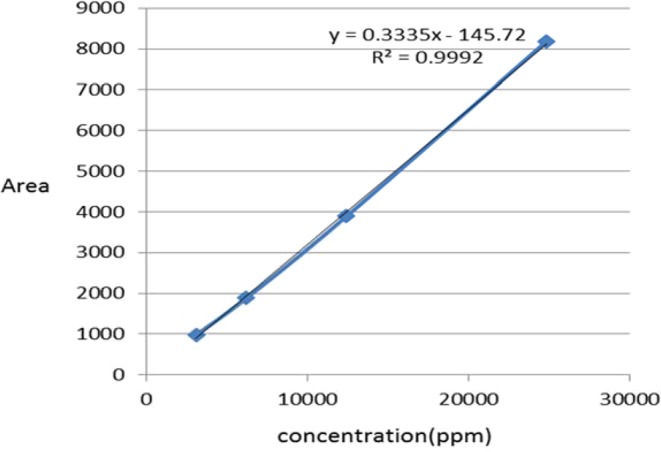
Calibration curve for propionic acid.

Figure 2 shows the calibration results for two Lactobacillus species in the range of 3100–24825 ppm. Propionic acid production showed an exceptional linear fit, with R^2^ values = 0.9992 for analytes.

The M4 strain produced 14576.11 ppm which was more than propionic acid produced by M2 strain (11697.3 ppm).

Table 4, demonstrated pH changes of Doogh samples during storage at three temperatures 4 °C, 25 °C and 37 °C, for 14, 21, and 60 days as an average of two replicates. pH changes of Doogh samples stored at 4 °C revealed the most noticeable change for from 4.41 to 3.97(% 0.44) until day-60 in samples inoculated with 10^8^ cfu/ml of M4 strain, and the lowest one was for control sample from 4.48 to 4.20 (% 0.28). At 25 °C, the highest variation was observed for the sample inoculated with 10^8^ cfu/ml of M4 strain with a change from 4.20 to 3.73 on day- 21 (0.47%), and the lowest one was related to control sample with a change of 4.35 to 3.98 (0.37%) indicates the least change during the storage period at 25 °C. Also, regarding pH changes of doogh samples during the storage period at 37 °C, the most significant reduction in pH was detected in sample inoculated with 10^8^ cfu/ml of M4 strain with a decline from 4.17 to 3.6 on day-14 (0.57%), and the lowest one was for control (4.35 to 3.95) (0.40%) indicates the least change during the storage period at 37 °C. In the present study, the decline of pH in doogh samples over storage period happened due to organic acid (i.e. lactic acid) accumulation which produced by yogurt starter culture as secondary metabolites.

**Table 4:** pH changes of Doogh samples produced during storage at 4, 25, 37 °C. pH changes of Doogh samples produced during storage at 4 °C.

sample	Production Day (day-0)	Day 10	Day 20	Day 30	Day 40	Day 50	Day 60
Control	4.48^a^	4.41^bc^	0.028 ± 4.32^gh^	0.014 ± 4.31^gh^	0.014 ± 4.29^hi^	0.028 ± 4.22^kl^	0.035 ± 4.20^mn^
${M}_{2}{10}^{6}$	4.40^cd^	0.014 ± 4.34^fg^	0.028 ± 4.27^ig^	0.021 ± 4.26^gk^	0.007 ± 4.25^jk^	0.007 ± 4.12^mn^	0.014 ± 4.09^n^
${M}_{2}{10}^{8}$	4.48^ab^	0.021 ± 4.37^ef^	0.028 ± 4.31^gh^	0.014 ± 4.28^hi^	0.014 ± 4.26^jk^	0.021 ± 4.18^lm^	0.028 ± 4.12^mn^
${M}_{4}{10}^{6}$	4.39^de^	0.014 ± 4.35^de^	0.021 ± 4.32^gh^	4.30^hi^	0.014 ± 4.28^hi^	0.014 ± 4.19^lm^	0.014 ± 4.05^mn^
${M}_{4}{10}^{8}$	4.41^bc^	0.021 ± 4.33^gh^	0.021 ± 4.29^hi^	0.021 ± 4.27^hi^	0.007 ± 4.25^jk^	0.035 ± 4.12^mn^	0.035 ± 3.97°
**pH changes of Doogh samples produced during storage at 25 °C**							
Sample	day-0	Day 7	Day 14	Day 21			
Control	4.35^a^	0.021 ± 4.13^cd^	0.035 ± 4.07^fg^	0.014 ± 3.98^g^			
${M}_{2}{10}^{6}$	4.24^ab^	0.014 ± 4.16^bc^	0.021 ± 3.96^ef^	0.021 ± 3.83^fg^			
${M}_{2}{10}^{8}$	4.23^bc^	0.021 ± 4.11^cd^	0.028 ± 3.84^fg^	0.028 ± 3.76^g^			
${M}_{4}{10}^{6}$	4.26^ab^	0.176 ± 4.17^bc^	0.028 ± 3.94^fg^	0.014 ± 3.84^g^			
${M}_{4}{10}^{8}$	4.20^bc^	0.042 ± 4.05^de^	0.014 ± 3.86^fg^	0.049 ± 3.73^g^			
**pH changes of samples produced during the storage period at 37 °C**.							
Sample	day-0	Day 7	Day 14				
Control	4.35^a^	0.028 ± 4.20^cd^	0.028 ± 3.95^e^				
${M}_{2}{10}^{6}$	4.23^bc^	0.014 ± 3.87^g^	0.014 ± 3.75^hi^				
${M}_{2}{10}^{8}$	4.20^cd^	0.007 ± 4.02^de^	0.021 ± 3.7^g^				
${M}_{4}{10}^{6}$	4.26^b^	0.021 ± 3.88^h^	0.028 ± 3.78^i^				
${M}_{4}{10}^{8}$	4.17^de^	0.042 ± 3.88^f^	0.014± 3.6^h^				

Non-similar alphabets in each column represent significant difference of mean values at the level of α = 95% or (p-value < 0.05).

Microbiological analysis on Mold and yeast, Coliform, *Staphylococcus aureus* and *Escherichia coli* counts revealed that the number of mold and yeast in the control sample on day 50 exceeded the standard and was unacceptable (Fig. 1) during 60 days of storage at 4 °C. However, mold and yeast count reached 0.3 × 10^2^ in Doogh sample inoculated with 10^6^ CFU/ml of strain M4 (*Lb. brevis*) and stored until day- 60. Comparing with the standard limit of mold and yeast according to Iranian Standard for Doogh (No. 2453), which equates to 100 CFU/ml, our findings for mold and yeast count (0.3 × 10^2^ CFU/ml) was less than standard limit (100 CFU/ml). Propionic acid slow down the growth of fungi at low pHs and, at pH below 4.5, affects the fungal membrane and disables them^26^.

**Figure 3:**
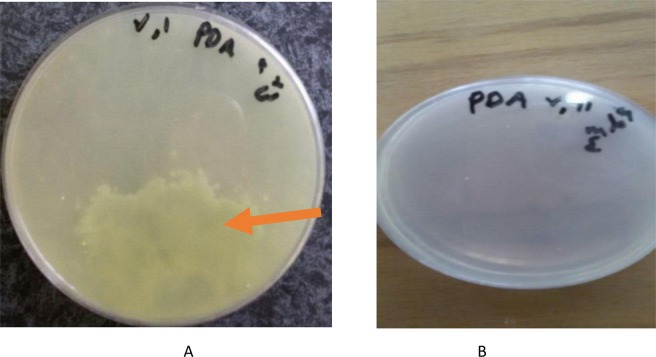
Control sample after 50 days of storage at 4 °C (**A**), sample inoculated with M4 10^6^ CFU/ml after 60 days of storage at 4 °C (**B**). PDA: Potato Dextrose Agar.

As shown in Fig. 3(A,B), sample inoculated with 10^6^ CFU/ml (6 Log CFU) of strain M4 (*Lb. brevis*) was remained uncontaminated at 4 °C for 60 days (0.3 × 10^2^ CFU/ml below the standard limit, 100 CFU/ml) (Standard No. 2453), which approves the antimicrobial activity of aforementioned strain. As shown in Fig. 2(A), control sample of Doogh was contaminated with mold and yeast on day −50.

Mold and yeast analysis was conducted during the 21 days of storage at 25 °C. Mold and yeast was uncountable in control sample on day-14 but sample inoculated with M4(10^6^) did not show any contamination with mold and yeast until day-21.

Microbiological analysis was done on mold and yeast, Coliform, *Staphylococcus aureus*, and *E. coli* during 14 days of storage at 37 °C. A control sample was positive in terms of mold and yeast and unacceptable on day-7 but sample inoculated with 10^6^ CFU/ml of strain M4 was negative in terms of mold and yeast. Thus it is regarded as acceptable after 14 days of storage. *Escherichia coli* was negative at all three storage temperatures. pH reduction of doogh samples during storage can inhibit many pathogenic bacteria. Because of the low pH of doogh (less than 4.5), pathogenic bacteria such as *Escherichia coli* do not have the ability to survive for a long time. Studies have shown that inoculated doogh samples with *E.coli* experienced a reduction from 7 log CFU/ml to 1 log CFU/ml after 2 weeks of storage at refrigerated temperature^2^. Mildew and yeasts are common spoilage microorganisms of food, such as fermented milk products, cheese, bread, as well as forage and alfalfa^27^. Yeasts are not involved in the fermentation process during yogurt production, but they are a major cause of spoilage of the final product^28^.

**Figure 4:**
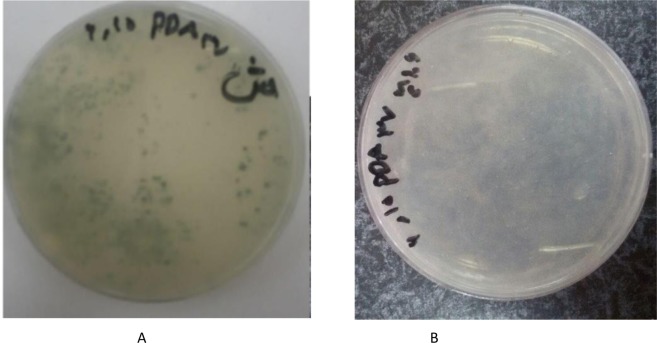
Control sample on day-7 at 37 °C (**A**) and sample inoculated with 10^6^CFU/ml of strain M4 after 14 days of storage at 37 °C. PAD: Potato Dextrose Agar.

As shown in Fig. 4 (A and B), control sample of day 7 was contaminated with mold and yeast, but the sample inoculated with 10^6^ cfu/ml of M4 strain, after 14 days of storage, evaluation of mold and yeast, and other bacteria responsible for the spoilage was negative.

### Sensory analysis results

Sensory analysis data imply that Doogh samples inoculated with *Lb. brevis* strains gained better sensory scores than the control sample.

**Figure 5:**
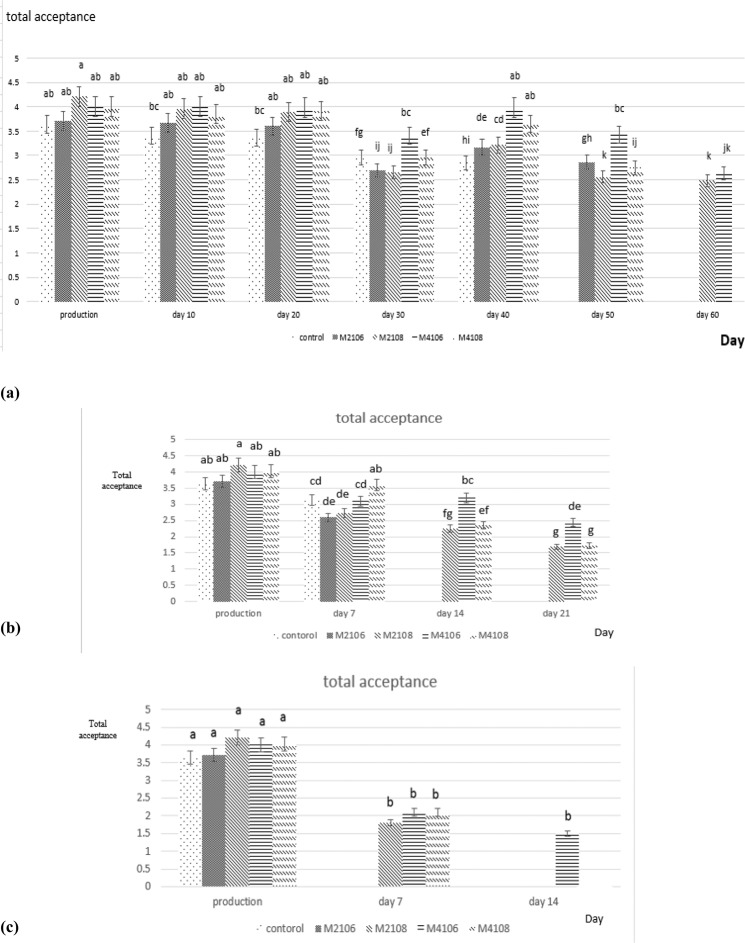
Comparison of the mean sensory analysis results (total acceptance) of Doogh samples produced during storage at 4 °C (**a**), at 25 °C (**b**) and at 37 °C (**c**).

Higher sensory scores in examined samples is probably due to production of metabolites such as diacetyl, acetic acid and other organic acid which can influence the aroma of dairy products. Doogh sample inoculated with strain M4(10^6^ cfu/ml) gained the highest score for texture in all three storage temperatures (4, 25 and 37 °C) and the lowest texture score was related to control. Evaluation of the flavor attribute in samples stored at 4 °C up to day- 20 revealed that, the highset score was related to Doogh sample inoculated with strain M4 (10^8^ cfu/ml) and from day-20 until day-60, was specified to sample inoculated with M4 (10^6^ cfu/ml). However, samples stored at 25 °C, until day-7, the highest flavor score was related to Doogh inoculated with strain M4 (10^8^ cfu/ml). From day-7 onward, Doogh sample inoculated with strain M4 (10^6^ cfu/ml) was scored more suitable which was probably due to noticeable increase of acidity in doogh inoculated with strain M4 (10^8^ cfu/ml) and the lowest score was recorded for control sample. In 37 °C, on production day, the highest flavor score was attributed to doogh samples incorporated with strain M4 (10^8^ cfu/ml). On day-7 and day-14 of storage, doogh containing strain M4(10^6^ cfu/ml) gained the highest flavor score. Color index for samples stored at all three temperatures (4, 25 and 37 °C), showed that the most score was attributed to doogh sample inoculated with strain M2(10^8^ cfu/ml) at the production day and from the production day onward, the highest color score was related to sample inoculated with strain M4 (10^8^ and 10^6^ cfu/ml) and the lowest score was related to control sample. Finally, the highest score for total acceptance was seen in Doogh sample inoculated with M2 (10^8^ cfu/ml) on production day at all three temperatures (Fig. 5). From production day onwards, the highest total acceptance score was seen in samples with strain M4 (10^6^ cfu/ml).

## Discussion

Results revealed that the greater inhibitory capability in the Agar Spot method compared with the well diffusion assay when the cell-free extract was used. Therefore, it is concluded that the presence of the isolates or colony-associated compounds, in the Agar spot method, enhances the anti-yeast activity. In the Agar Spot method, more colony-associated antimicrobial compounds such as fatty acids and hydrogen peroxide, are responsible for creating inhibition halo in the solid medium^29^.

Since propionic acid is an antifungal compound, the strong anti-yeast activity of the M4 strain might be partly attributed to the production of organic acids, especially propionic acid^30^. The performance of these two strains as the strongest anti-yeast species is probably due to the production of more organic acids, such as propionic acid. Antifungal activity of some strains of *L. plantarum* has been reported by^31^, which is related to organic acids, cyclic dipeptides and low molecular mass metabolites that produced by the bacteria. Moreover, Magnusson *et al*. 2003, have tested the antifungal activity of a large number of Lactobacillus isolates from a different environment. A number of isolates was showed strong growth inhibition against *A. fumigatus*^32^.

A logical corrolation and positive relationship was seen between data obtained for concentration of propionic acid and sensory propertie. For texture attribute, this preference or superiority in samples inoculated with M2 and M4 strains is owing to metabolites produced with these two strains. It seems that factors affecting on texture changes in primary stages of storage is different from final stages. Moisture and pH are among factors which influence on texture changes during primary storage stages^33^. Based on Table 4, pH changes of Doogh samples during storage in three temperatures showed declining trend which can be one of the reasons for decreasing the texture scores of Doogh samples. Azizi *et al*., (2017) reported that *Lb.brevis* strains M2 and M4 produced antimicrobial and bacteriocin-like compounds which affect on sensory attributes of the product^14^. Lactic acid bacteria produce exopolysacharide and other metabolite cause noticeable increase in viscosity and slimy structure which increases with inoculation of bacterium so that samples produced with *Lb.brevis* strains gained the highest texture score^2^.

Although different organic acids have been recognized in fermented milk products such as yogurt, but only some of them have noticeable influence on flavor of product, so that the most important aromatic flavor is acetaldehyde in yogurt^34^.

Ekinci and Gurel (2008) indicated that incorporation of Propionibacterium spp. in yogurt production resulted in acetaldehyde increase on first day^35^.

Farhadi *et al*., (2012) found that fermented dairy beverage with maximum content of propionic acid 1.2(%w/w) had gained the highest score for flavor and total acceptability^36^.

Since the propionic acid concentration was higher in sample inoculated with strain M4 (14576.11 ppm) than the one inoculated with strain M2 (11697.3 ppm), the highest score is related to this sample (sample inoculated with strain M4).

In order to evaluate the anti-yeast activity of twenty strains of lactic acid bacteria isolated from Iranian traditional raw milk cheeses (Lighvan and Motal), two selected strains namely M2 and M4 previously identified as *Lb. brevis* subspecies showed the highest anti-yeast activity in culture-based methods (agar spot and well diffusion assay). Doogh samples inoculated with 10^6^ CFU/ml of strain M4 also exhibited the best quality according to microbial evaluation in all three storage temperatures (4, 25 and 37°C). No mold and yeast growth was detected on day-14 at 37°C.

Since M4 sample in which *Lb. brevis* strain has been inoculated at the highest level of 10^8^ CFU/ml, therefore, it seems logical that the maximum pH reduction has occurred in this sample. However, both strains of M2 and M4, have been identified as *Lb. brevis*. It is obvious that the acid production capacity and pH reduction is a strain-dependent phenomenon, which reflects interspecies variations. Many metabolites play a role in the antimicrobial and antifungal effects of lactic acid bacteria, which organic acids are one of these factors. As the results confirmed the acidity in inoculated samples was more than in control sample which explained their antifungal activity. On the other hand, contrary to the present study, some researchers have shown addition of *Lactobacillus rhamnosus* did not affect yogurt pH compared to addition of yogurt starter. Therefore, differences in pH variations of different samples can be attributed to different bacterial species and strains^37^.

Based on the results of the pH-changes during storage, at 4, 25, and 37 °C, the most noticeable changes were detected at 37°C and the least ones at 4 °C, indicating that with increasing of temperature, acid production increases, implying pH reduction associated with the organic acids accumulation including lactic acid, acetic acid and propionic acid^38^ produced by lactobacilli.

Sodium propionate and ammonium have the same influence on yeasts and mildew at low pH^39^. Lactic acid is a major and main metabolite of lactic acid bacteria that reduces pH and inhibits many microorganisms. Heterofermentative LABs, such as some lactobacilli, produce relatively large amounts of acetic acid in the presence of foreign electron receptors, while propionic acid is produced at a negligible amount. Propionic and acetic acids inhibit the absorption of amino acid^38^.

LAB produce active peptides with biological activity and protein complexes which indicate antibacterial mechanism against close- related species^40,41^. Compounds with antimicrobial properties may be detected in a broad range of Gram-positive and Gram-negative bacteria. Among aforementioned compounds, those produced by LAB are highly appreciated due to their preservative nature in the food processing^42^. Nisin, as the first authorized bacteriocin, have been applying in the food industry in 46 countries leastwise^43^. The prime mode of action for peptide bacteriocin to be mentioned infraction of the cell membrane in target microorganisms^44^.

Propionic and acetic acid, as short-chain organic acids, secreted by LAB are popularly used by food manufacturers as antimicrobial additives or acidulants in a diversity of food products^45^. The capability of yeasts as food-spoilage and food-borne organisms in dairy goods is connected with their nutritional requirements, certain enzymatic activities and the ability to grow at low temperatures, low pH values, low water activities and high salt concentrations^46^.

## Supplementary information

Supplementary information

## Data Availability

All data are completely accessible without limitation.

## References

[1] Naidu AS, Bidlack WR, Clemens RA (1999). Probiotic spectra of lactic acid bacteria (LAB). Crit Rev Food Sci Nutr..

[2] Kocak, C. and Avsar, Y.K. Ayran: *Microbiology and Technology*. In: Yildiz, F. (Ed.). Development and Manufacture of Yogurt and Functional Dairy Products. Boca Raton, U.S: 123–141(CRC Press, 2009).

[3] Sayevand HR (2018). Bacterial Diversity in Traditional Doogh in Comparison to Industrial Doogh. Curr Microbiol.

[4] Pitt, J. J. & Hocking, A. D. Fungi and Food Spoilage. 2nd ed. Gaithersburg, MD: (Aspen, 1999).

[5] Sweeney M, Dobson A (1998). Mycotoxin production by Aspergillus, Fusarium and Penicillium species. Int. J. Food Microbiol..

[6] Canganella F (1998). Survival of undesirable micro-organisms in fruit yogurts during storage at different temperatures. Food Microbiol..

[7] Gülmez M, Güven A, Sezer Ç, Duman B (2003). Evaluation of microbiological and chemical quality of ayran samples marketed in Kars and Ankara cities in Turkey. Kafkas Univ. Vet. Med. J..

[8] Tamang, J. P. & Fleet, G. H. Yeasts diversity in fermented foods and beverages. In: Satyanarayana, T. and Kunze, G. (Eds). Yeast Biotechnology: Diversity and Applications, Dordrecht Netherlands. 169–198, 10.1007/978-1-4020-8292-4_9 (Springer, 2009).

[9] Sen, E. & Küplülü, O. Determination of agreement to Turkish Food Codex of unpackaged ayran consumed in Ankara. Etlik Veteriner Mikrobiyoloji Dergisi (*J of Etlik Vet. Microbiol*. **15**, 55–60 (2004) (in Turkish).

[10] Ashurst, P. R. *Chemistry and Technology of Soft Drinks and Fruit Juices*. (2nd ed). 57–62 (Blackwell Publishing, 2005).

[11] Stiles ME (1996). Biopreservation by lactic acid bacteria. Antonie van Leeuwenhoek..

[12] Lindgren SE, Dobrogosz WJ (1990). Antagonistic activities of lactic acid bacteria in food and feed fermentations. FEMS Microbiol. Rev..

[13] Carr FJ, Chill D, Maida N (2002). The lactic acid bacteria: a literature survey. Crit. Rev. in Microbiol..

[14] Azizi F, Habibi Najafi MB, Edalatian Dovom MR (2017). The biodiversity of Lactobacillus spp. from Iranian raw milk Motal cheese and antibacterial evaluation based on bacteriocin-encoding genes. AMB Express.

[15] Edalatian MR (2012). Microbial diversity of the traditional Iranian cheeses Lighvan and Koozeh, as revealed by polyphasic culturing and culture-independent approaches. Dairy Sci. and Technol..

[16] Rouse S, Harnett D, Vaughan A, Sinderen Dvan (2008). Lactic acid bacteria with potential to eliminate fungal spoilage in foods. J. of Appl. Microbiol..

[17] Yang EJ, Chang HC (2010). Purification of a new antifungal compound produced by *Lactobacillus plantarum* AF1 isolated from kimchi. Int. J. of Food Microbiol..

[18] Scherer R (2012). Validation of a HPLC method for simultaneous determination of main organic acids in fruits and juices. Food Chem..

[19] Crowley S, Mahony J, Van Sinderen D (2012). Comparative analysis of two antifungal *Lactobacillus plantarum* isolates and their application as bio protectants in refrigerated foods. J. of Appl. Microbiol..

[20] Standards Iran. Doogh – Specifications and test method (No. 2453-2008) (2008).

[21] Standards Iran. Milk and milk products –Enumeration of coliforms part 2 (No. 5486-2-2002). Retrieved from http://standard.isiri.gov.ir/ (2002).

[22] Standards Iran. Microbiology of food and animal feeding Stuffs - Horizontal method for the (Staphylococcus aureus and other species) Enumeration of coagulates Positive staphylococci, Part 3: Detection and MPN technique for low numbers (No. 6806-3-2003). Retrieved from http://standard.isiri.gov.ir/ (2003).

[23] Standards Iran. Milk and Milk Products-Enumeration of Presumptive Escherichia coli-Most probable number (MPN) (No. 5234- 2016). Retrieved from http://standard.isiri.gov.ir/ (2016).

[24] Standards Iran. General guidance for enumeration of yeasts and moulds colony count technique at 25°C (No. 997-1993). Retrieved from http://standard.isiri.gov.ir/ (1993).

[25] Standards Iran. General Method for Sensory Evaluation of Dairy Products (No. 4691-2000). Retrieved from http://standard.isiri.gov.ir/ (2000).

[26] Woolford MK (1984). The antimicrobial spectra of organic compounds with respect to their potential as hay preservatives. Grass Forage Sci..

[27] Bonestroo MH, Dewit JC, Kusters BJM, Rombouts FM (1993). Inhibition of the growth of yeasts in fermented salads. Int. J. Food Microbiol..

[28] Fleet GH (1990). Yeasts in dairy products. A review. J. Appl. Microbiol..

[29] Alegría Á, Delgado S, Roces C, López B, Mayo B (2010). Bacteriocins produced by wild *Lactococcus lactis* strains isolated from traditional, starter-free cheeses made of raw milk. Int. J. Food Microbiol..

[30] Schnu¨rer J, Magnusson J (2005). Antifungal lactic acid bacteria as bio preservatives. Trends Food Sci. Technol..

[31] Lavermicocca P (2000). Purification and characterization of novel antifungal compounds from the sourdough *Lactobacillus plantarum* strains 21B. Appl. Environ. Microbiol..

[32] Magnusson J, Storm K, Roos S, Sjogren J, Schnurer J (2003). Broad and complex antifungal activity among environmental isolates of lactic acid bacteria. FEMS Microbiol. Lett..

[33] Lawrence R, Creamer L, Gilles J (1987). Texture development during cheese ripening. J. Dairy Sci..

[34] Law BA (1981). The formation of aroma and flavor compounds in fermented dairy products. Dairy Sci. Abstr..

[35] Ekinci FY, Gurel M (2008). Effect of using propionic acid bacteria as an adjunct culture in yogurt production. J. Dairy Sci..

[36] Farhadi S (2012). Effect of incubation temperature and inoculation ratio of starter culture on propionic acid production in dairy beverage fermented with Propionibacterium. Iranian J. of Nutr. Sci. & Food Technol..

[37] Delavenne E, Ismail R, Pawtowski A, Miunier G, Barbier G (2012). Assessment of lactobacilli strains as yogurt bio protective cultures. Food Control..

[38] Eklund, T. Organic acids and esters. In G. W. Gould (Ed.). Mechanisms of action of food preservation procedures. 161–200 (Elsevier. New York, 1989).

[39] Woolford MK (1984). The antimicrobial spectra of some salts of organic acids and glutaraldehyde in respect to their potential as silage additives. Grass Forage Sci..

[40] Tagg JR, Dajani AS, Wannamaker LW (1976). Bacteriocins of Gram-positive bacteria. Bacteriol. Rev..

[41] Klaenhammer TR (1988). Bacteriocins of lactic acid bacteria. Biochimie..

[42] Ross RP, Morgan S, Hill C (2002). Preservation and fermentation: past, present, and future. Int. J. Food Microbiol..

[43] Delves-Broughton J, Blackburn P, Evans RJ, Hugenholtz J (1996). Applications of the bacteriocin, nisin. Antonie van Leeuwenhoek..

[44] Hechard Y, Sahl HG (2002). Mode of action of modified and unmodified bacteriocins from Gram-positive bacteria. Biochimie..

[45] Davidson, P.M. & Juneja, V.K. Antimicrobial agents. In: Branen AL, Davidson PM, Salminen, S. (Eds.), Food Additives, New York, 1–9 (Marcel Dekker Inc, 1990).

[46] Fleet G, Mian MA (1987). The occurrence and growth of yeasts in dairy products. Int. J. Food Microbiol..

