# Supplementary material for: Determination of the anti-yeast activity of Lactobacillus spp. isolated from traditional Iranian cheeses in vitro and in yogurt drink (Doogh)
Source: Sci Rep. 2020 Apr 14;10:6291. doi: 10.1038/s41598-020-63142-0 (PMC7156417; doi:10.1038/s41598-020-63142-0)
Supplement: Supplementary file 1 — Supplementary information [file 41598_2020_63142_MOESM1_ESM.docx]

**Supplementary file**

**Title Page**

**Determination of the anti-yeast activity of Lactobacillus spp. isolated from traditional Iranian cheeses *in vitro* and in yogurt drink (Doogh)**

Saeid Afzali^1^, Mohammad Reza Edalatian Dovom*^2^, Mohammad Bagher Habibi Najafi^3^, Mostafa Mazaheri Tehrani^4^

1. Graduated MSc student, Food Science and Technology Department, Agriculture Faculty, Ferdowsi University of Mashhad (FUM), Mashhad, Iran.
2. Associate Professor, Food Science and Technology Department, Agriculture Faculty, Ferdowsi University of Mashhad (FUM), Mashhad, Iran. Corresponding Author, Email: [edalatian@um.ac.ir](mailto:edalatian@um.ac.ir) , Tel: +985138805774.
3. Professor, Food Science and Technology Department, Agriculture Faculty, Ferdowsi University of Mashhad (FUM), Mashhad, Iran.
4. Professor, Food Science and Technology Department, Agriculture Faculty, Ferdowsi University of Mashhad (FUM), Mashhad, Iran.

Table 1: pH changes of samples produced during the storage period at 37 ° C.

Table 2. Microbial results of Doogh during storage at 4 ° C.

Table 3. Microbial results of Doogh during storage at 25 ° C.

Table 4. Microbial results of dough samples produced during storage at 37 ° C.

**Table 1: pH changes of samples produced during the storage period at 37 ° C.**

| Day 14 | Day 7 | Production Day | Sample |
| --- | --- | --- | --- |
| 0.028±3.95e | 0.028±4.20cd | 4.35a | Control |
| 0.014±3.75hi | 0.014±3.87g | 4.23bc | $M_{2}{10}^{6}$ |
| 0.021±3.7g | 0.007 ±4.02de | 4.20cd | $M_{2}{10}^{8}$ |
| 0.028±3.78i | 0.021±3.88h | 4.26b | $M_{4}{10}^{6}$ |
| 0.014±3.6h | 0.042±3.88f | 4.17de | $M_{4}{10}^{8}$ |

Non-similar alphabets in each column represent significant difference of mean values at the level of α = 5% or (p-value <0.05).

**Table 2. Microbial results of Doogh during storage at 4 ° C.**

| M_4_10^8^ | M_4_10^6^ | M_2_10^8^ | M_2_10^6^ | Control | Day |  |
| --- | --- | --- | --- | --- | --- | --- |
| ND | ND | ND | ND | ND* | Production Day |  |
| ND | ND | ND | ND | ND | Day 10 |  |
| ND | ND | ND | ND | ND | Day 20 |  |
| ND | ND | ND | ND | ND | Day 30 | mold and yeast |
| ND | ND | ND | ND | ND | Day 40 |  |
| 4.7×10^2^ | - | 0.6×10^2^ | 0.4×10^2^ | mold | Day 50 |  |
| mold | 0.3×10^2^ | 1.5×10^2^ | mold | mold | Day 60 |  |
| ND | ND | ND | ND | ND | Production Day |  |
| ND | ND | ND | ND | ND | Day 10 |  |
| ND | ND | ND | ND | ND | Day 20 |  |
| ND | ND | ND | ND | ND | Day 30 | Coliform |
| ND | ND | ND | ND | ND | Day 40 |  |
| ND | ND | ND | ND | ND | Day 50 |  |
| mold | ND | ND | 0.1×10^2^ | ND | Day 60 |  |
| ND | ND | ND | ND | ND | Production Day |  |
| ND | ND | ND | ND | ND | Day 10 |  |
| ND | ND | ND | ND | ND | Day 20 |  |
| ND | ND | ND | ND | ND | Day 30 | *Staphylococcus aureus* |
| ND | ND | ND | ND | ND | Day 40 |  |
| ND | ND | ND | ND | 0.1×10^2^ | Day 50 |  |
| ND | ND | ND | ND | uncountable | Day 60 |  |
| ND | ND | ND | ND | ND | Production Day |  |
| ND | ND | ND | ND | ND | Day 10 |  |
| ND | ND | ND | ND | ND | Day 20 | *Escherichia coli* |
| ND | ND | ND | ND | ND | Day 30 |  |
| ND | ND | ND | ND | ND | Day 40 |  |
| ND | ND | ND | ND | ND | Day 50 |  |
| ND | ND | ND | ND | ND | Day 60 |  |

The microbial results of the produced dough samples during the storage period at 4 ° C as an average of two replicates.

ND: not detected.

**Table 3. Microbial results of Doogh during storage at 25 ° C.**

| M_4_10^8^ | M_4_10^6^ | M_2_10^8^ | M_2_10^6^ | Control | Day |  |
| --- | --- | --- | --- | --- | --- | --- |
| ND | ND | ND | ND | ND | Production |  |
| ND | ND | ND | ND | ND | Day 7 | mold and yeast |
| ND | ND | 1.1×10^3^ | ND | non-countable | Day 14 |  |
| 8× 10^2^ | ND | non-countable | 1× 10^2^ | non-countable | Day 21 |  |
| ND | ND | ND | ND | ND | Production |  |
| ND | ND | ND | ND | ND | Day 7 | Coliform |
| ND | ND | ND | ND | ND | Day 14 |  |
| ND | ND | 1× 10^2^ | ND | 1.5× 10^2^ | Day 21 |  |
| ND | ND | ND | ND | ND | Production |  |
| ND | ND | ND | ND | ND | Day 7 | *Staphylococcus aureus* |
| ND | ND | ND | ND | ND | Day 14 |  |
| ND | ND | ND | ND | 4× 10^2^ | Day 21 |  |
| ND | ND | ND | ND | ND | Production |  |
| ND | ND | ND | ND | ND | Day 7 | *Escherichia coli* |
| ND | ND | ND | ND | ND | Day 14 |  |
| ND | ND | ND | ND | ND | Day 21 |  |

The microbial results of the produced dough samples during the storage period at 25 ° C were averaged over two times.

**Table 4. Microbial results of dough samples produced during storage at 37 ° C.**

| M_4_10^8^ | M_4_10^6^ | M_2_10^8^ | M_2_10^6^ | Control | Day |  |
| --- | --- | --- | --- | --- | --- | --- |
| ND | ND | ND | ND | ND | Production Day (0) |  |
| ND | ND | 1.2×10^3^ | ND | mold | Day 7 | Mold and yeast |
| mold | ND | mold | mold | mold | Day 14 |  |
| ND | ND | ND | ND | ND | Production |  |
| ND | ND | ND | ND | ND | Day 7 | Coliform |
| ND | ND | ND | ND | ND | Day 14 |  |
| ND | ND | ND | ND | ND | Production |  |
| ND | ND | ND | ND | ND | Day 7 | *Staphylococcus aureus* |
| ND | ND | 1.2×10^3^ | ND | 4× 10^2^ | Day 14 |  |
| ND | ND | ND | ND | ND | Production |  |
| ND | ND | ND | ND | ND | Day 7 | *Escherichia coli* |
| ND | ND | ND | ND | ND | Day 14 |  |

The microbial results of the produced doogh samples during the storage period at 37°C are mean of two replicates.
